# Overactive bladder induces transient hypertension

**DOI:** 10.1186/s13104-018-3317-6

**Published:** 2018-03-27

**Authors:** Kazumasa Torimoto, Yoshihiro Matsumoto, Daisuke Gotoh, Yosuke Morizawa, Makito Miyake, Shoji Samma, Nobumichi Tanaka, Akihide Hirayama, Kiyohide Fujimoto

**Affiliations:** 1 Department of Urology, Nara Medical University, 840 Shijo-cho, Kashihara, Nara 634-8522 Japan; 2 Department of Urology, Hoshigaoka Medical Center, 4-8-1 Hoshigaoka, Hirakata, Osaka 573-8511 Japan; 3 Department of Urology, Nara Prefecture General Medical Center, 1-30-1 Hiramatsu, Nara, Nara 631-0846 Japan; 4 Department of Urology, Kindai University Nara Hospital, 1248-1 Otoda-cho, Ikoma, Nara 630-0227 Japan

**Keywords:** Hypertension, Overactive bladder, Transurethral resection of prostate, Micturition

## Abstract

**Objectives:**

Several studies have shown the relationship between lower urinary tract symptoms and autonomic imbalance. We investigated the relationship between detrusor overactivity (DO) or urgency, and transient increase in blood pressure as a type of hypertension related to sympathetic hyperactivity. Study 1: we enrolled 14 male patients with DO and 10 without DO. We measured the overactive bladder symptom score (OABSS) and blood pressure during cystometry. Study 2: we enrolled 14 men patients with overactive bladder (OAB) and 8 without OAB. We measured OABSS and blood pressure using a 24-h ambulatory device.

**Results:**

Study 1: the mean systolic pressure was significantly higher at urgency or SDV than at the other measurement points in the DO group (161.3 ± 23.2 vs. 134.5 ± 16.3, 137.8 ± 15.3, or 139.5 ± 14.8 mmHg). Study 2: the mean systolic pressure was significantly higher at the measurement points before micturition than at the points unrelated to micturition in the OAB group (159.7 ± 24.9 vs. 124.9 ± 13.8 mmHg). In conclusion, DO or urgency induces a transient increase of blood pressure, suggesting that OAB induces a type of hypertension before micturition.

## Introduction

Metabolic syndrome, in particular, diabetes and hypertension, are related to the clinical progression of benign prostatic hyperplasia [1]. Hypertension is related to higher international prostate symptom scores and larger prostate volumes [2]. A previous cohort study found that the prevalence of hypertension was higher in subjects with overactive bladder (OAB) than those without OAB [3]. Male patients with hypertension had more severe lower urinary symptoms (LUTS) than those without hypertension [4]. These findings suggest that hypertension results in more mechanical or functional LUTS. Moreover, sympathetic hyperactivity leads to hypertension.

In female patients, several studies on the relationship between OAB and sympathetic hyperactivity have been reported [5, 6]. Takami reported that anticholinergic (imidafenacin 0.2 mg/day) treatment for female nocturia due to OAB resulted in improvement in nocturnal hypertension and morning hypertension; he concluded that the treatment also inhibited sympathetic hyperactivity [7]. This suggests that OAB may induce autonomic imbalance, especially sympathetic hyperactivity. In this study, we investigated the relationship between detrusor overactivity (DO) or urgency and transient increase in blood pressure as a type of hypertension related to sympathetic hyperactivity in male patients.

## Main text

### Methods

#### Study design

We conducted an exploratory study at two hospitals in Japan from July 2009 to March 2011. All patients provided written informed consent prior to enrollment. The study was performed in accordance with the Declaration of Helsinki, and approval was obtained from the institutional review board at Nara Medical University hospital.

##### Study 1

We enrolled male patients with any LUTS. Exclusion criteria were histories of lower urinary tract neoplasms, vesical stones, symptomatic urinary tract infection, and neurogenic bladder. We evaluated their storage symptoms using the overactive bladder symptom score (OABSS) and measured blood pressure with a sphygmomanometer during cystometry at first desire to void (FDV), normal desire to void (NDV), strong desire to void (SDV) or urgency, and after micturition. We performed cystometry with patients in sitting position and sphygmomanometry with a manchette placed on the left upper arm. We calculated the bladder outlet obstruction index (BOOI) using the formura: BOOI = Pdet at Qmax − 2 (Qmax) [8], and the bladder contractility index (BCI) using the formula: BCI = Pdet at Qmax + 5 (Qmax) [9]. We did not calculate the study size before the enrollment because this is an exploratory study and no information was available to set the size according to previous studies.

##### Study 2

We enrolled male patients with any LUTS. Exclusion criteria were histories of lower urinary tract neoplasms, vesical stones, symptomatic urinary tract infection, and neurogenic bladder. We evaluated their storage symptoms using OABSS and measured blood pressure with a 24-hour ambulatory blood pressure monitoring device (FB-270^®^, Fukuda Denshi, Tokyo, Japan). Diagnosis of OAB was made using OABSS, which evaluates daytime frequency (score, 0–2), nocturia (score, 0–3), urgency (score, 0–5), and urgency urinary incontinence (score, 0–5) over a 1-week recall period. Patients were diagnosed as having OAB if the score for Question 3 regarding urgency yielded was ≥ 2 points and if the total score was ≥ 3 points [10]. We set the device to measure blood pressure every 30 min in the daytime from 6:00 to 22:00 and every 60 min in the nighttime from 22:00 to 6:00 in order to decrease the influence on sleep. We also asked the patients to measure blood pressure before micturition and record micturition time charts. We did not calculate the study size before the enrollment because this is an exploratory study and no information was available to set the size according to previous studies.

#### Statistical methods

The data were checked for Gaussian distribution and appropriate two-sided tests were chosen. Specific statistical tests are listed on corresponding tables. All statistical analyses were performed using Prism version 7.03. A p value of < 0.05 was considered statistically significant.

## Results

### Study 1

#### Patient characteristics

We enrolled 14 patients with DO and 10 patients without DO. The mean OABSS, BOOI, and BCI were significantly higher in the group with DO than in the group without DO. Bladder outlet obstruction was considered to exist in 11 patients (78.6%) in the group with DO and 3 patients (30.0%) in the group without DO (Table 1).

**Table 1:** Patient characteristics in study 1

Variable	With DO	Without DO
Age		
Mean ± SD (years)	72.5 ± 6.3	72.3 ± 11.3
OABSS		
Mean ± SD	8.3 ± 2.4	3.8 ± 3.2*
BOOI		
Mean ± SD	75.9 ± 55.4	23.6 ± 21.9*
≥ 40, n (%)	11 (78.6)	3 (30)*
BCI		
Mean ± SD	130 ± 39.0	96.6 ± 34.2*

The Mann–Whitney U test was used to compare age, OABSS, BOOI, and BCI

The Fisher’s exact test was used to compare the proportion of patients with BOOI ≥ 40

*DO* detrusor overactivity, *OABSS* overactive bladder symptom score, *BOOI* bladder outlet obstruction index, *BCI* bladder contractility index

* p < 0.05 versus “with DO”

#### Blood pressure and pulse

The mean systolic and diastolic pressure were significantly higher at urgency or SDV than before infusion, FDV, or NDV, and after micturition in the group with DO. The mean pulse did not change during cystometry. Meanwhile, the mean systolic and diastolic pressure, and the mean pulse did not change through cystometry in the group without DO (Table 2).

**Table 2:** Blood pressure and pulse during cystometry in Study 1

	Before infusion	FDV or NDV	Urgency or SDV	After micturition
With DO				
Systolic pressure (mmHg)	134.5 ± 16.3	137.8 ± 15.3	161.3 ± 23.2*	139.5 ± 14.8
Diastolic pressure (mmHg)	80.9 ± 11.0	84.3 ± 12.5	92.1 ± 13.7*	84.1 ± 12.5
Pulse (per minute)	78.8 ± 15.6	81.4 ± 16.2	82.8 ± 16.7	81.5 ± 13.4
Without DO				
Systolic pressure (mmHg)	131.5 ± 18.3	135.5 ± 16.9	135.9 ± 11.7*	131.9 ± 18.6
Diastolic pressure (mmHg)	79.0 ± 14.6	80.9 ± 13.7	83.4 ± 11.9*	80.0 ± 11.1
Pulse (per minute)	71.6 ± 7.9	71.9 ± 8.6	72.6 ± 7.1	73.2 ± 12.7

The Friedman test with a post hoc test (Dunn’s multiple comparison test) to compare blood pressure and pulse during cystometry

The values are expressed as mean ± SD

*DO* detrusor overactivity, *FDV* first desire to void, *NDV* normal desire to void, *SDV* strong desire to void

* p < 0.05 versus Before infusion, FDV or NDV and After micturition

### Study 2

#### Patient characteristic

We enrolled 14 patients with OAB and 8 patients without OAB. The mean age of the patients with OAB and those without OAB were 72.9 ± 4.5 and 69.4 ± 6.4 years, respectively. The mean OABSS was significantly higher in the group with OAB than in the group without OAB (8.9 ± 2.2 vs. 2.1 ± 0.4, p < 0.05). The number of patients with hypertension, who all received hypertensive drugs, was 6 (43%) in the group with OAB and 4 (50%) in the group without OAB. Nobody had central nerve disease which may influence storage function (Table 3).

**Table 3:** Patient characteristics in Study 2

Characteristic	OAB (n = 14)	Non OAB (n = 8)
Age		
Mean ± SD (years)	72.9 ± 4.5	69.4 ± 6.4
OABSS		
Mean ± SD	8.9 ± 2.2	2.1 ± 0.4*
Hypertension		
n (%)	6 (43%)	4 (50%)

The Mann–Whitney U test was used to compare age and OABSS

The Fisher’s exact test was used to compare the proportion of patients with hypertension

*OABSS* overactive bladder symptom score

* p < 0.05 versus OAB

#### Blood pressure and pulse

The mean systolic and diastolic pressure were significantly higher at the measurement points before micturition than at the points unrelated to micturition in the group with OAB. The mean pulse did not differ significantly over time. Meanwhile, the mean systolic pressure was also significantly higher at the measurement points before micturition than at the measurement points unrelated to micturition in the group without OAB. The mean diastolic pressure and pulse were not significantly different. The mean systolic pressure was significantly higher in the group with OAB than in the group without OAB. Mean systolic and diastolic pressure at the measurement points unrelated to micturition was not significantly different for the group with OAB and the group without OAB (Table 4). Four of the patients with OAB, who were enrolled in the study 2, suffered from LUTS and hoped to receive transurethral resection of the prostate (TURP). The mean OABSS and systolic pressure before micturition decreased significantly 6 months after TURP compared to before TURP (1.3 ± 2.5 vs. 10.3 ± 1.9, p < 0.05; 129.4 ± 15.7 vs. 170.4 ± 31.8 mmHg, p < 0.05) (Wilcoxon signed rank test).

**Table 4:** Blood pressure and pulse measured using a 24-h ambulatory blood pressure monitoring device in Study 2

	Before micturition	Unrelated to micturition
With OAB		
Systolic pressure (mmHg)	159.7 ± 24.9	124.9 ± 13.8*
Diastolic pressure (mmHg)	93.2 ± 11.8	79.4 ± 10.6*
Pulse (per minute)	71.1 ± 11.6	70.7 ± 10.5
Without OAB		
Systolic pressure (mmHg)	123.6 ± 14.1^†^	116.2 ± 17.1*
Diastolic pressure (mmHg)	75.3 ± 13.2	72.5 ± 11.1
Pulse (per minute)	69.7 ± 9.8	68.1 ± 8.9

The Mann–Whitney U test was used to compare blood pressure at the measurement points before micturition and at the points unrelated to micturition

The values are expressed as mean ± SD

*OAB* overactive bladder

* p < 0.05 versus before micturition

^†^ p < 0.05 versus “with OAB”

## Discussion

Several studies on the relationship between OAB and autonomic imbalance (sympathetic hyperactivity) have been reported. These studies measured heart rate variability using electrocardiogram, which noninvasively detects autonomic imbalances [11, 12]. These studies showed that OAB is related to an imbalance of the autonomic nervous system in female patients [5] and that urgency, urgency urinary incontinence, and detrusor overactivity are related to sympathetic hyperactivity in female patients [6]. In neurogenic OAB, neurogenerative diseases such as Parkinson’s disease, multiple system atrophy, and multiple sclerosis impair the autonomic nerve system and cause LUTS, including OAB [13–15]. Accordingly, it has been proposed that the autonomic imbalances may induce OAB, even in patients with idiopathic OAB, which is most frequently encountered in the clinic. We have an opposing view regarding this hypothesis, in that OAB itself may induce sympathetic hyperactivity. As previously stated, Takami reported that an anticholinergic agent, imidafenacin, treatment for OAB inhibited sympathetic hyperactivity [7].

We demonstrated that DO or urgency, which usually coexists with DO, induces a transient steep increase in blood pressure in male patients. In general, sympathetic stimuli contract the arteries, and blood pressure and pulse increases. Thus, DO or urgency induces transient sympathetic hyperactivity. The reason for selecting male patients was that we needed to perform pressure flow study to check BOO before TURP. However, we believe that the same phenomenon would occur in female patients with OAB. Although “hypertension” is transient, the increase in blood pressure and the frequency of “hypertension” should be higher in patients with more severe OAB. The systolic pressure actually increases to more than 200 mmHg when a patient with severe BOO shows DO during a cystometrogram. Frequent occurrence of strong urgency and transient hypertension may cause patients stress and impair autonomic balance. Therefore, OAB is not just a local urinary continence and frequency issue, but sometimes it affects the global cardiovascular system.

The mechanisms which increases blood pressure during DO or urgency remain unknown. We believe that autonomic dysreflexia (AD) in patients with spinal cord injury is a similar phenomenon. AD is an acute syndrome of excessive, uncontrolled, sympathetic output with potentially serious consequences that can occur in patients who have had an injury to the spinal cord (generally at or above the sixth thoracic neurologic level) [16]. From a urology viewpoint, bladder distention, especially DO occurrence, produces an afferent impulse that generates a generalized sympathetic response, which in turn results in vasoconstriction below the neurologic lesion. The activation of the C-fiber afferent nerve is believed to be related to lower urinary tract dysfunction, such as DO in the chronic phase of spinal cord injury [17]. If the spinal cord is intact, descending central inhibitory pathways respond to the increase in blood pressure and modulate the sympathetic activity. However, if the spinal cord is injured, inhibitory signals do not reach the sympathetic chain [16]. One clinical study demonstrated that the C-fiber afferent nerve is activated in patients with non-neurogenic OAB [18]. Even in OAB patients without spinal cord injury, urgency afferent signals via the C-fiber might directly stimulate the sympathetic chain and cause widespread vasoconstriction, which cannot be suppressed due to some OAB-specific pathology.

In conclusion, DO or urgency induces a transient increase in blood pressure, suggesting that OAB induces a type of hypertension. Hypertension can be relieved after patients undergo transurethral resection of the prostate.

## Limitations

The goal of this study was to estimate the influence of frequent transient hypertension due to OAB on the whole body. However, we did not measure autonomic imbalance directly or secretion of catecholamine. We did not check the symptoms of the hypertension, such as headache, sweating, and uneasiness. We also investigate whether DO or urgency- related hypertension could be relieved if OAB is treated well. We showed that a small number of patients (n = 4) for which TURP relieved OAB and hypertension. We do not think the numbers are sufficient. We need to perform further well-designed prospective studies based on the present study to confirm this phenomenon and global influence.

For Table 4, those parameters were “within-subject”. However, the two events (“Before micturition” and “Unrelated to micturition”) randomly occurred for 24 h and did not line up with time unlike typical repeated measures. The parameters were not normally distributed. That is why we chose the Mann–Whitney U-test as unpaired nonparametric test. We acknowledge that a two-way ANOVA with the factors OAB and timepoint could also have been used.

## Abbreviations

OAB: overactive bladder
LUTS: lower urinary tract symptom
DO: detrusor overactivity
OABSS: overactive bladder symptom score
FDV: first desire to void
NDV: normal desire to void
SDV: strong desire to void
BOOI: bladder outlet obstruction index
BCI: bladder contractility index
BOO: bladder outlet obstruction
TURP: transurethral resection of the prostate
AD: autonomic dysreflexia
